# Cardiotoxic Effects Produced by Omeprazole and Methylene Blue in an Animal Model of Cardiac Ischemia and Reperfusion and Potential Implications for the Pharmacological Strategy for Vasoplegic Syndrome

**DOI:** 10.3390/biomedicines12030582

**Published:** 2024-03-06

**Authors:** Erisvaldo Amarante de Araújo, Fernando Sabia Tallo, Alex Sandro Felisberto Oliveira, Gustavo Saad Silva El Toghlobi, Rafael Augusto Arantes, Rafael Balsimelli, Bruno Kehrwald-Balsimelli, Bianca Lorayne de Almeida Viana, Fernanda Sakata Matuda, Lucas Antonio Duarte Nicolau, Jand Venes Rolim Medeiros, Adriano Caixeta, Murched Omar Taha, Walter José Gomes, Afonso Caricati-Neto, Francisco Sandro Menezes-Rodrigues

**Affiliations:** 1 Postgraduate Program in Cardiology, Universidade Federal de São Paulo (UNIFESP), São Paulo 04024-000, SP, Brazil; biomedipatologia@icloud.com (E.A.d.A.); acaixeta@me.com (A.C.); 2 Discipline of Urgency and Emergency Care, Universidade Federal de São Paulo (UNIFESP), São Paulo 04024-000, SP, Brazil; 3 Postgraduate Program in Interdisciplinary Surgical Science, Universidade Federal de São Paulo (UNIFESP), São Paulo 04024-002, SP, Brazil; alex.oliveira@unifesp.br (A.S.F.O.); taha@uol.com.br (M.O.T.); 4 Department of Medicine, Universidade Santo Amaro (UNISA), São Paulo 04829-300, SP, Brazil; gustavosaad98@gmail.com (G.S.S.E.T.); r.arantes.medico@gmail.com (R.A.A.); rafaelbalsimelli2002@gmail.com (R.B.); brunobalsimelli@estudante.unisa.br (B.K.-B.); biancaalmeida.viana@gmail.com (B.L.d.A.V.); 5 Department of Medicine, Universidade Nove de Julho (UNINOVE), São Paulo 01504-001, SP, Brazil; fernanda.sakata.m@gmail.com; 6 Research Center on Biodiversity and Biotechnology, Universidade Federal do Delta do Parnaíba (UFDPar), Parnaíba 64202-020, PI, Brazil; lucasnicolau@ufpi.edu.br (L.A.D.N.); jandvenes@ufpi.edu.br (J.V.R.M.); 7 Discipline of Cardiovascular Surgery, Universidade Federal de São Paulo (UNIFESP), São Paulo 04024-000, SP, Brazil; wjgomes1012@gmail.com; 8 Department of Pharmacology, Universidade Federal de São Paulo (UNIFESP), São Paulo 04023-062, SP, Brazil; caricatineto@gmail.com

**Keywords:** vasoplegic syndrome, cardiac ischemia–reperfusion, methylene blue, omeprazole, cardiac arrhythmias

## Abstract

Defined as systemic hypotension caused by intense vasodilation due to the loss of systemic vascular resistance, vasoplegic syndrome (VS) is associated with elevated morbidity and mortality in humans. Although vasopressors such as norepinephrine and vasopressin are the first-choice drugs for VS treatment, several other drugs such as methylene blue (MB) can be used as adjuvant therapy including rescue therapy. To develop new pharmacological strategies to reduce the risk of VS, we investigated the effects of treatments with MB (2 mg/kg/IV), omeprazole (OME, 10 mg/kg/IV), and their combination in an animal model of cardiac ischemia–reperfusion (CIR). The ventricular arrhythmia (VA), atrioventricular block (AVB), and lethality (LET) incidence rates caused by CIR (evaluated via ECG) and serum levels of the cardiac lesion biomarkers creatine kinase–MB (CK-MB) and troponin I (TnI) in adult rats pretreated with saline solution 0.9% and submitted to CIR (SS + CIR group) were compared to those pretreated with MB (MB + CIR group), OME (OME + CIR group), or the MB + OME combination (MB + OME + CIR group). The AVB and LET incidence rates in the MB + CIR (100%), OME + CIR (100%), and MB + OME + CIR (100%) groups were significantly higher compared to the SS + CIR group (60%). The serum level of CK-MB in these groups were also significantly higher compared to the SS + CIR group, demonstrating that the treatments before CIR with MB, OME, and MB + OME produced similar effects in relation to cardiac function and the occurrence of lesions. These results demonstrate that the treatment of animals subjected to the CIR protocol with OME produced the same effects promoted by the treatment with MB, which may suggest the possibility of using OME alone or in combination with MB in medical clinics in treatment of VS.

## 1. Introduction

A well-known complication following cardiac surgery, either with or without cardiopulmonary bypass (CPB), is vasoplegic syndrome (VS), which carries a high risk of perioperative morbidity and death. Its occurrence range in cardiac surgery patients following CPB has been reported by some authors to be between 9% and 44% in the subset of patients with predisposing characteristics [1,2]. VS is manifested by significant systemic hypotension associated with continuously high cardiac output and low systemic vascular resistance, yet poor quality organ perfusion, although vasopressors are needed to sustain the blood pressure for end-organ perfusion [1,2].

The mechanism linking cardiac surgery with CPB to VS is complex and depends on the type of surgery performed and several patient-specific factors. The broad base immune response, complement activation elicited by surgical trauma, ischemia–reperfusion injuries to the heart and lungs, blood transfusions, and blood exposure to the foreign surfaces of the CPB circuit are most likely linked to post-CPB vasoplegia. Increased levels of oxygen-free radicals, endothelins, nitric oxide (NO), platelet-activating factors, thromboxane A2, prostaglandins, various cytokines, and other vasoactive substances are the outcomes of these events. Vascular relaxation is determined by the relative plasma concentrations of the endogenous mediators described above. Furthermore, these variables contribute to the emergence of a systemic inflammatory response syndrome (SIRS), which exacerbates the dilatation of the generalized arteries [3,4,5,6]. 

Uncontrolled vasodilation and vascular hyporesponsiveness to fluid resuscitation and endogenous vasoconstrictors are the precursors of refractory VS, which results in a breakdown of the physiological regulating mechanics of vascular tone. Non-catecholaminergic vasopressors such as thiamine, ascorbic acid, corticosteroid, terlipressin, angiotensin II, hydroxocobalamin, vasopressin, and methylene blue (MB) have been utilized recently to improve VS and restore vascular tone. Their impact on the mortality benefits, however, is currently unclear. Despite recent improvements in treatment, the mortality rates are still very high, mainly due to multiple organ failure, particularly acute kidney injury [3,4,5,6].

It is well established that the vascular tone and systemic arterial pressure are physiologically regulated by several vasoconstrictor and vasodilator factors, including norepinephrine released from sympathetic nerves and NO released from endothelial cells. Endothelial NO exerts a crucial role in the control of vascular tone and vasodilation. NO is synthesized by the enzymatic action of NO synthase (NOS) on the amino acid L-arginine. NO-mediated vasodilation is resultant from the activation of guanylate cyclase (GC) and consequent increase in intracellular levels of cGMP in vascular cells, which inhibits Ca^2+^ influx and activates K^+^ channels, reducing vascular tone. MB has been used in the treatment of VS due its vasodilatory actions mediated by the inhibition of inducible NOS (iNOS) and consequent reduction in NO synthesis [7,8,9,10]. Additionally, it has been observed that the proton pump inhibitor (PPI) agents, such as omeprazole (OME), decrease the phosphorylation of endothelial NOS (eNOS) brought on by bradykinin (BK) [7,8,9,10]. This implies that PPIs decrease the availability of NO, most likely through a mechanism that has already been proposed (such as a decrease in eNOS expression or an increase in intracellular asymmetrical dimethylarginine levels) [11,12]. However, the effects of MB and OME in reducing the risk of VS are still little known.

To develop new pharmacological strategies to reduce the risk of VS associated with cardiac surgery and reduce its morbidity and mortality in humans, we investigated the effects of treatments with MB (2 mg/kg/IV), omeprazole (OME, 10 mg/kg/IV), and their combination in an animal model of cardiac ischemia–reperfusion (CIR). The ventricular arrhythmia (VA), atrioventricular block (AVB), and lethality (LET) incidence rates caused by CIR (evaluated by ECG) and serum levels of cardiac lesion biomarkers creatine kinase–MB (CK-MB) and troponin I (TnI) in adult rats pretreated with a saline solution (0.9%) and submitted to CIR (SS + CIR group) were compared to those pretreated with MB (MB + CIR group), OME (OME + CIR group), and the MB + OME combination (MB + OME + CIR group).

## 2. Materials and Methods

### 2.1. Animals

The animals utilized in this study (Wistar rats, male, weighing from 280 to 320 g) were maintained under standard conditions of nutrition, hydration, temperature (21 ± 2 °C), light (12:12 h light/dark cycle), and humidity, and in accordance with normalization protocols approved by the Ethics Committee of the Escola Paulista de Medicina EPM)/Universidade Federal de São Paulo (UNIFESP). All experimental protocols used in this study were approved by the Ethics Committee of the EPM/UNIFESP (UNIFESP #9447210317 and 7323080822).

### 2.2. Induction of Cardiac Ischemia and Reperfusion (CIR)

To replicate an animal model of AMI in the lab, rats underwent surgical procedures following the protocol that our research group had previously published [13,14]. To induce unconsciousness, the rats were initially given intraperitoneal injections of xylazine (10 mg/kg), ketamine (100 mg/kg), and tramadol (2 mg/kg). The rats were placed in the supine position on a surgical platform that was heated by a thermal blanket after being given anesthesia. A rectal thermometer was used to regularly check the temperature, which was kept at 37.5 °C. Using a respiratory pump from Insight^®^ (EFF 312—Insight Equipamentos Cientificos, Ribeirão Preto, Brazil), the animals were kept on mechanical ventilation. Initially, a venous access procedure was carried out via the femoral vein, involving the implantation of a catheter to deliver the medication at the suitable moment. The animals were then put through mechanical ventilation using room air with a tidal volume of roughly 6 mL/kg of body weight and a respiratory frequency of 90 cycles per minute, after orotracheal intubation.

A left thoracotomy was carried out between the fourth and fifth intercostal spaces following the trichotomy. A 4-0 suture (4/0 braided silk suture coupled to a 10-mm micropoint reverse cutting needle; Ethicon K-890H, Raritan, NJ, USA) was passed approximately 2 mm from the origin, between the edge of the left atrium and the sulcus of the pulmonary artery, after the pericardium was broken. This allowed the heart to be externalized through lateral compression of the chest. The chest was then promptly reopened, and the heart was swiftly returned to the thoracic cavity. The two ends of the nylon thread were fed into a cylindrical polypropylene tube, which was utilized to create ischemia, in order to accomplish the coronary ligation [15,16].

Following a stabilization period of fifteen minutes, the coronary artery was covered by the tube, the nylon thread was removed, and the tube and nylon thread were secured using Kelly forceps. All that needed to be done to accomplish reperfusion was to separate this arrangement and take out the nylon thread and tube. The tourniquet was withdrawn to allow for 75 min of coronary after 10 min of myocardial ischemia. The procedures for the sham group were the same as those previously described, although instead of performing a coronary ligation the nylon thread was only slipped under the left coronary artery. As a result, ischemia and reperfusion were not caused. ECG monitoring was continued throughout the duration of the experiment following surgery. As outlined below, various experimental protocols were used [17,18,19].

### 2.3. Evaluation of Cardiac Activity during CIR

Using a procedure that our research group had previously published [17,18,19], an electrocardiogram (ECG) analysis was utilized to analyze the cardiac activity during CIR. Using this high-resolution methodology, several researchers evaluated the cardioprotective effects of calcium channel blockers and other drugs on the incidence rates of cardiac arrhythmias (VA and AVB) and lethality (LET) owing to CIR. Prior to the stabilization phase the ECG was first recorded for 15 min, then during the 75 min ischemia and reperfusion protocols it was recorded for 10 min. The ECG was recorded using a biopotential amplifier and needle electrodes that were subcutaneously placed into the limbs. Changes in the ECG caused by CIR (increases in the R wave and ST segment) were used to confirm the effective coronary artery [17,18,19].

The ECG was recorded using a biopotential amplifier and needle electrodes that were subcutaneously implanted into the limbs. The coronary artery had been successfully blocked via surgery, as demonstrated by the ECG anomalies (increases in R wave and ST segment) caused by CIR. The body temperature was maintained at 37.5 °C using a heated operating table and the appropriate heating lamps, and the temperature was frequently checked with a rectal thermometer. The ECG data were processed using a computer system that included AqDAnalysis 7 software and AqDados 7.02 hardware (Lynx Tecnologia Ltd., São Paulo, Brazil) [17,18,19]. With this method, we were able to track not only the incidence rates of CIR-induced VA, AVB, and LET but also heart rates. VA was the classification given to torsades, atrial fibrillation, and ventricular fibrillation [17,18,19].

### 2.4. Biochemical Determination of Serum Levels of Cardiac Lesions Biomarkers

After the experiment had been carried out or the animal had died, the serum CK-MB and TnI levels were determined using the methodology described in our previous studies [20]. The rats that made it through the entire 75-min CIR therapy provided the blood samples. After being extracted from the abdominal aorta and placed in siliconized tubes, these 4–5 mL samples were centrifuged for 40 min at 2500 rpm and 5 °C. The supernatant was removed and stored at −20 °C for the enzymatic detection of CK-MB and TnI at 340 nm. For this, a kinetic UV test kit was obtained from Vida Biotecnologia, located in Belo Horizonte, Brazil [20].

### 2.5. Drugs Used in the Study

The MB and OME utilized in the study were obtained from Sigma-Aldrich, Brazil. The animals were treated via intravenous (IV) administration through the left femoral vein with MB (Sigma Aldrich, Saint Louis, MO, United States) at 2 mg/kg and OME (Sigma Aldrich, Saint Louis, MO, United States) at 10 mg/kg before CIR to evaluate the incidence rates of VA, AVB, and LET caused by CIR using an ECG analysis. The following experimental groups were created from the animals used in this study:

(1) SS + CIR group (*n* = 20): Rats treated with a saline solution (SS) and submitted to CIR;
(2) MB + CIR group (*n* = 12): Rats treated with MB (2 mg/kg, IV) and submitted to CIR;
(3) OME + CIR group (*n* = 12): Rats treated with OME (10 mg/kg, IV) and submitted to CIR;
(4) MB + OME + CIR group (*n* = 12): Rats treated with MB (2 mg/kg, IV) plus OME (10 mg/kg, IV) and submitted to CIR.

### 2.6. Analysis of Statistics

The incidence rates of VA, AVB, and LET expressed as percentages were analyzed using the Prism 8.0 program (GraphPad, Boston, MA, USA) and statistically analyzed using Fisher’s exact test [19]. The serum concentrations of the cardiac lesion biomarkers CK-MB and TnI expressed as the mean ± the standard error of the mean (SEM) were submitted to an analysis of variance (ANOVA) test followed by Tukey’s post-test using the Prism 8.0 program (GraphPad, USA) [19]. The results were considered statistically significant when *p* < 0.05 [19].

## 3. Results

### 3.1. Effects of MB and OME on the Incidence Rates of VA, AVB, and LET Induced by CIR

**Figure 1:**
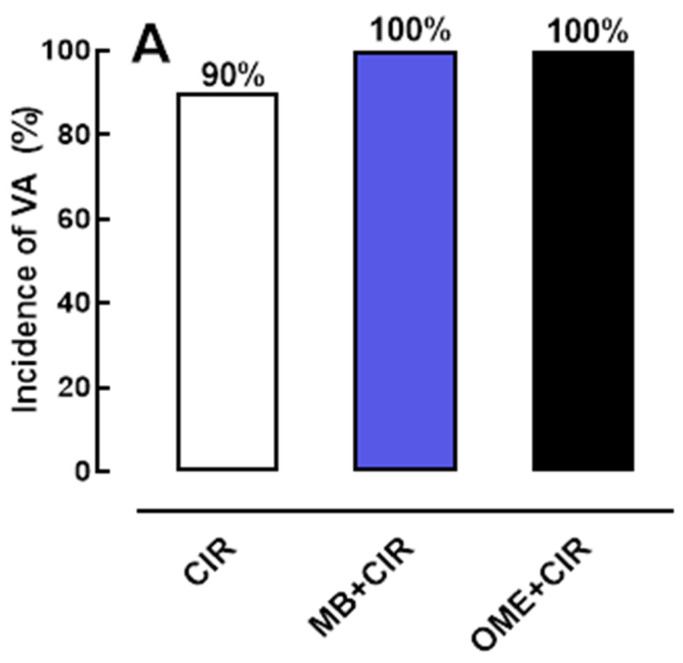

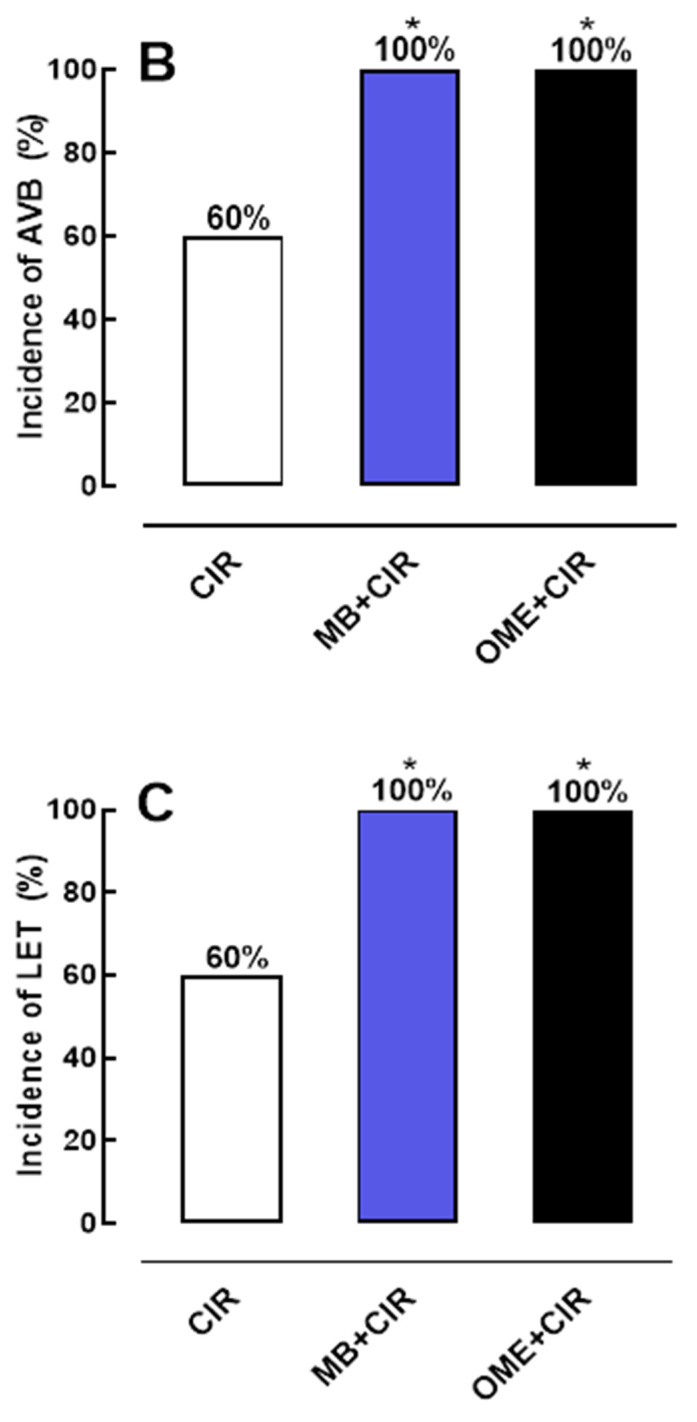
Histograms representing the (**A**) incidence rates of ventricular arrhythmias (VA), (**B**) atrioventricular block (AVB), and (**C**) lethality (LET) in the rats pretreated with methylene blue (MB) at 2 mg/kg/IV (*n* = 12), omeprazole (OME) at 10 mg/kg/IV (*n* = 12), or saline solution 0.9% (SS) (*n* = 20) and submitted to cardiac ischemia and reperfusion (CIR). The results are expressed as ratios and the statistical analysis was performed using Fisher’s exact test. Note: * *p* < 0.05 statistically different compared to the SS + CIR group.

Figure 1 shows that the AVB and LET incidence rates but not the VA rates in the MB + CIR (100%) and OME + CIR (100%) groups were statistically different when compared to the SS + CIR group (60%), indicating that treatment with MB at 2 mg/kg/IV or OME at 10 mg/kg/IV before CIR increased the AVB and LET incidence rates induced by CIR. Similar results were obtained when MB at 2 mg/kg/IV and OME at 10 mg/kg/IV were administrated before CIR. The AVB and LET incidence rates in the MB + OME CIR group (100%) were statistically different when compared to the SS + CIR group (60%), indicating that the treatment with MB at 2 mg/kg/IV plus OME at 10 mg/kg/IV before CIR increased the AVB and LET incidence rates induced by CIR. 

### 3.2. Effects of the Treatments with MB and OME on the Serum Levels of CK-MB and TnI in Animals Submitted to CIR

Table 1 shows that the serum levels of CK-MB in the MB + CIR and OME + CIR groups were statistically different when compared to the SS + CIR group, indicating that treatment with MB at 2 mg/kg/IV or OME at 10 mg/kg/IV before CIR increased the serum levels of CK-MB in rats submitted to CIR.

Table 1 also shows that the serum levels of TnI in the MB + CIR and OME + CIR groups were not statistically different when compared to the SS + CIR group, indicating that treatment with MB at 2 mg/kg/IV or OME at 10 mg/kg/IV before CIR did not change the serum levels of TnI in the rats submitted to CIR.

## 4. Discussion

VS is a well-known complication following cardiac surgery, either with or without CPB, which represents an important perioperative risk factor associated with elevated morbidity and mortality rates in patients submitted to cardiac surgery. Although several classes of vasopressor drugs have been proposed to restore vascular tone and systemic arterial pressure in patients with VS, the pharmacological treatment of this syndrome remains under investigation. In order to develop new pharmacological strategies to reduce the risk of VS associated with cardiac surgery and reduce its morbidity and mortality in humans, in the present work we investigated the effects of treatments with MB (2 mg/kg), OME (10 mg/kg), and their combination in an animal model of cardiac ischemia–reperfusion (CIR). The present study shows that the AVB and LET incidence rates in the MB + CIR (100%), OME + CIR (100%), and MB + OME + CIR (100%) groups were significantly higher compared to the SS + CIR group (60%). In addition, the serum levels of CK-MB and TnI in these groups were also significantly higher compared to the SS + CIR group. These results suggest that treatment with the isolated or combined use of MB and OME could be effective and safe in patients with VS due to a reduction in NO bioavailability, which restores vascular tone and systemic arterial pressure in patients with VS. This study supports the notion that treatment with the isolated or combined use of MB and OME could be an effective and safe way to reduce the risk of VS in patients undergoing cardiac surgery. 

The goal of early postoperative VS therapy should be to identify the issue when hypotension, poor SVR, normal or supranormal cardiac output, and fluid unresponsiveness are present. The goal of caregiving for a patient at risk of postoperative ventilator-associated pneumonia is to intervene before shock sets in. However, many risk variables, including some that are intrinsic parts of the surgical procedure, cannot be changed in the immediate preoperative period. Vasopressor therapy should ideally be started after cardiac function optimization and fluid resuscitation have been completed. Next, the treatment of VS involves the use of catecholaminergic drugs with alpha-adrenergic activity (phenylephrine, norepinephrine (NE), dopamine, and epinephrine), non-catecholaminergic drugs (arginine-vasopressin and angiotensin II), and moderators of NO (MB, hydroxocobalamin, vitamin C, thiamine, and corticosteroids) [3,4,5,6,21,22,23,24,25,26,27,28,29,30,31].

Additionally, due to neurohypophysis store depletion and release during extended CPB and surgery, the plasma levels of arginine vasopressin (AVP) are low to normal. Because AVP can reduce NO synthesis and mitigate its vasomotor effects, it plays a pivotal role in the pathophysiology of VS. The length of CPB determines the relative or absolute lack of AVP levels, as well as the elevated SIRS, and these factors are attributed to VS. Thus, decreased vasopressin plasma levels, increased NO synthesis, and SIRS are implicated as the leading causes of VS following cardiac surgery with prolonged CPB [21,22,23,24,25,26,27,28,29]. The excessive complexity in the cellular mechanisms involved in VS following CPB and the involvement of inflammatory cytokines and iNOS are probably the primary factors in the improper vasodilation associated with vasoplegia. The amount of NO that iNOS produces raises the level of vascular cyclic guanosine monophosphate (cGMP), which causes vasodilation. The length of the CPB is directly correlated with the amount of iNOS in the plasma and the severity of VS [21,22,23,24,25,26,27,28,29,30,31].

Another pathophysiologic role for NO in vasoplegia is as a K^+^ channel activator, especially in K_ATP_ channels [15]. Furthermore, even in the presence of elevated catecholamine levels in these cells, vasoconstriction will not transpire due to the deactivation of voltage-activated Ca^2+^ channels (VACC) during CPB caused by lactic acidosis, intracellular acidosis, and reduced adenosine triphosphate (ATP) levels. Additionally, the cytoplasmic Ca^2+^ levels are reduced and the vasoconstriction impact is blunted by opening the Ca^2+^-sensitive K^+^ channels (K_Ca_) and the channels of K_ATP_, which is a potent intracellular vasodilator mediator [1,15,16,17]. Hydrogen sulfide is another pathophysiologic mediator, which in some conditions, such as inflammation, directly activates and hyperpolarizes K_ATP_ channels, hence lowering vascular tone [32]. This mechanism has similarities to the previously known NO-mediated pathway of vasoplegia. Its synergistic impact with NO may account for a minor amount of its vasodilatory effects [25,26,27,28,29,30,31,32,33].

Although pharmacological therapies have been proposed and used for the stabilization and recovery of patients with VS, the morbidity and mortality rates remain very high. Therefore, a new class of pharmacological agents used in isolation or combination appears to hold promise in the treatment of VS. Pantoprazole and OME, known as PPIs, can be administered intravenously to inhibit the vasodilatory response due to a reduction in NO bioavailability, which restores the vascular tone and systemic arterial pressure in patients with VS. PPIs have drawn a lot of attention for the anti-cancer effects they have through apoptosis induction and anti-inflammatory actions [23,24,25,26,27,28]. Nevertheless, the dosages of these PPIs used in basic and clinical research to investigate the anti-cancer effects are higher than those used in clinical settings to treat gastroesophageal reflux disease [34,35,36,37,38,39,40]. The vascular physiology may be affected by high dosages of PPIs, as some basic research studies have suggested [40,41], although no studies have looked at the impact of PPIs on endothelial Ca^2+^ signaling or the generation of endothelium-derived relaxing factor (EDRF) [41,42,43].

Recent advances in basic research have identified the pleiotropic effects of PPIs. Fako et al. [44] demonstrated that PPIs are effective inhibitors of human fatty acid synthase’s thioesterase activity, which is linked to treatment resistance, a poor prognosis, and cancer cell survival. Indeed, OME inhibited thioesterase activity with a half-maximal inhibitory dose of 29.6 µM, while Bx3PC-3 cell survival occurred at a half-maximal concentration of 14.8 µM [32]. Consequently, even though the peak plasma concentration range of OME recorded during clinical usage is roughly 1–2 µM, the anti-cancer effects of high-dosage PPIs (i.e., 100 µM OME) have been assessed in recent basic and clinical studies.

To maintain vascular homeostasis, which includes blood coagulation, vascular permeability, and the synthesis of EDRF, endothelial cells are essential. Variations in intracellular Ca^2+^ concentrations ([Ca^2+^]i) are required for a number of endothelial activities. In endothelial cells, a crucial mechanism involved in [Ca^2+^]i regulation mediated by the endoplasmic reticulum (ER), known as store-operated calcium entry (SOCE), is typified by the ER’s Ca^2+^ mobilization and the extracellular space’s subsequent Ca^2+^ influx [45]. While a few fundamental studies have indicated that PPIs may affect the vascular physiology [31,32], very few have concentrated on how PPIs affect endothelial Ca^2+^ signaling.

The GPCR bradykinin receptor B2, which is found on the surfaces of endothelial cells, is activated by bradykinin (BK). The GPCR/PLC/IP3 pathway is triggered when BK stimulates the BK B2 receptor. This leads to an increase in Ca^2+^ release from the ER and the activation of store-operated Ca^2+^ channels (SOCC) [46,47,48]. By inhibiting the ER’s Ca^2+^-ATPase levels and passively reducing the ER’s Ca^2+^ levels, thapsigargin (TG) also stimulates SOCE [36,37,49,50,51]. The OME is able to lower TG-induced SOCE in primary cultured porcine aortic endothelial cells (PAECs), which is consistent with recent research that found that 100 µM OME prevented TG-induced SOCE in rat basophilic leukemia (RBL-1) mast cells [50]. 

Additionally, OME at a concentration of 100 µM lacks any pharmacological effects on ER Ca^2+^-ATPase, despite the possibility that it may partially reduce BK-induced Ca^2+^ release from the ER. Therefore, it is plausible that the GPCR/PLC/IP3 pathway and SOCC-related proteins were the two concurrent pathways by which OME inhibited the intracellular Ca^2+^ response. According to earlier observations, the interaction between the inhibitors and the protein known as Ca^2+^-release-activated Ca^2+^ modulator 1 is responsible for the inhibitory effects of a few SOCE inhibitors [28,29,30]. The GPCR/PLC/IP3 pathway may be impacted by OME or other PPIs, although this has not been mentioned in any published publications. Therefore, more research is required to precisely define OME’s inhibitory characteristics.

OME has also been reported to reduce the phosphorylation of eNOS caused by BK, suggesting that PPIs reduce the availability of NO, most likely via a previously postulated mechanism (such as an increase in intracellular asymmetrical dimethylarginine levels or a decrease in eNOS expression) [20,24,33]. Numerous external cues, including sphingosine 1-phosphate, BK, insulin, vascular endothelial growth factor, estrogen, and shear stress, might alter the activity of eNOS [35,36]. While BK-induced eNOS phosphorylation is mediated by calmodulin-dependent protein kinase II in a [Ca^2+^]i-dependent manner [48], insulin, estrogen, and vascular endothelial growth factor phosphorylate eNOS primarily via protein kinase B in a [Ca^2+^]i-independent manner [37,38]. Kamiya et al. demonstrated that OME inhibited BK-activated intracellular Ca^2+^ signaling, meaning the reduced eNOS phosphorylation is corroborated by these earlier results.

The regulation of eNOS activity in endothelial cells via the reciprocal phosphorylation of activator and inhibitor sites is one potential method [35]. Because Thr495 is constitutively phosphorylated, calmodulin binding is inhibited, which reduces the eNOS activity. Calmodulin’s binding to eNOS was enhanced by BK stimulation after phosphatase 1 dephosphorylated Thr495 [39]. According to a recent investigation, BK had no effect on the phosphorylation of Thr495 [38]. Additionally, only a minor increase in enzyme activity (less than a two-fold increase) is elicited by Ser1177 phosphorylation [27,31]. While the effect of OME on Thr495 phosphorylation was not evaluated, BK-induced NO generation may be influenced by the phosphorylation balance between Ser1177 and Thr495 after OME therapy. Increases in [Ca^2+^]i control the synthesis of prostaglandin I2 (PGI2), a significant vasodilator [32,33]. In endothelial cells, Ca^2+^-dependent phospholipase A2 must be activated by SOCE in order to convert membrane phospholipids into arachidonic acids, which are the building blocks of proteinoids [44].

As per earlier studies demonstrating the Ca^2+^-dependent synthesis of prostaglandins [52,53,54], the results demonstrated by Kamiya et al. [55] suggest that OME tended to reduce the production of 6-keto-PGF1α. Endothelial cells constantly generate PGI2 and NO. Furthermore, Kamiya et al. [55] also demonstrated that without BK or OME, the production rates of NO and PGI2 were 0.042 ± 0.032 µM/106 cells and 784.46 ± 212.45 pg/mL/106 cells, respectively, showing that both NO and PGI2 were produced at steady rates. For NO and PGI2, the effect of BK on these EDRF generation was increased by roughly 1.32 and 1.24 times, respectively, showing that the OME reduced the extra effect of BK on EDRF synthesis by roughly 0.96 times for NO and 1.03 times for PGI2. 

Based on the results from this study, as well as the data found in several articles published in respected and renowned scientific journals, we raise the possibility of using injectable PPI in association with MB in the treatment of patients with VS or even the use of a preventive approach in isolation from this class of drugs with the aim of attenuating or even abolishing the occurrence of VS in patients undergoing cardiac surgery with extracorporeal circulation. As a limitation of our study, we highlight that there was no extracorporeal circulation, which would certainly make the model more reliable and more similar to what happens in medical clinics, although we believe that the ischemia and reperfusion model used in this study provided us with important information to support our hypothesis.

## 5. Conclusions

Our results suggest that the treatment of animals subjected to the CIR protocol with OME produced the same effects promoted by treatment with MB, which may suggest the possibility of using OME alone or in combination with MB in medical clinics in the treatment of VS.

## Figures and Tables

**Table 1:** Serum concentrations of the cardiac lesion biomarkers creatine kinase–MB (CK-MB) and troponin I (TnI) in the rats pretreated with methylene blue (MB) at 2 mg/kg/IV, omeprazole (OME) at 10 mg/kg/IV, or saline solution 0.9% (SS) and submitted to cardiac ischemia and reperfusion (CIR).

Groups	CK-MB (U/L)	TnI I (ng/mL)
SS + CIR	2037 ± 117	0.200 ± 0.01
MB + CIR	2760 ± 292 *	0.200 ± 0.01
OME + CIR	2610 ± 245 *	0.200 ± 0.01

The results are expressed as the mean ± standard error of the mean (SEM) as obtained from 3 to 5 rats. The data were submitted to an analysis of variance (ANOVA) followed by Tukey’s post-test. SS + CIR group (*n* = 5); MB + CIR group (*n* = 3): OME + CIR group (*n* = 3). Note: * *p* < 0.05 statistically different compared to the SS + CIR group.

## Data Availability

The data will be available upon justified request and with the agreement of the authors.
